# The Prevention of Brain Metastases in Non-Small Cell Lung Cancer by Prophylactic Cranial Irradiation

**DOI:** 10.3389/fonc.2018.00241

**Published:** 2018-07-26

**Authors:** Willem J. A. Witlox, Bram L. T. Ramaekers, Jaap D. Zindler, Daniëlle B. P. Eekers, Judith G. M. van Loon, Lizza E. L. Hendriks, Anne-Marie C. Dingemans, Dirk K. M. De Ruysscher

**Affiliations:** ^1^Department of Clinical Epidemiology and Medical Technology Assessment, Maastricht University Medical Centre, Maastricht, Netherlands; ^2^Department of Radiation Oncology (Maastro Clinic), Maastricht University Medical Centre, GROW School of Oncology and Developmental Biology, Maastricht, Netherlands; ^3^Department of Pulmonology, Maastricht University Medical Centre, GROW School of Oncology and Developmental Biology, Maastricht, Netherlands; ^4^Radiation Oncology, KU Leuven, Leuven, Belgium

**Keywords:** non-small cell lung cancer, prophylactic cranial irradiation, brain metastases, toxicity, survival, quality of life

## Abstract

**Background:**

Non-small cell lung cancer (NSCLC) patients frequently develop brain metastases (BM), even though the initial imaging with brain CT or MRI was negative. Stage III patients have the highest risk to develop BM, with an incidence of approximately 30%. BM can lead to neurocognitive disorders, loss of quality of life (QoL), and they are the most important factors influencing patient’s overall survival (OS). Although a radical local treatment of BM may be possible with primary radiosurgery or after resection, the prognosis often remains poor. Preventing the development of BM through prophylactic cranial irradiation (PCI) may improve the outcome of these patients.

**Methods:**

Data from published randomized trials comparing PCI with non-PCI were sought using electronic database (PubMed) searching, hand searching, and by contacting experts. Trials were included if they considered a randomized comparison of PCI and non-PCI, enrolled NSCLC patients, excluded patients with recurrent or metastatic disease, and reported results on BM occurrence. Each randomized controlled trial (RCT) was assessed for methodological quality using the Cochrane collaboration’s tool for the assessment of risk of bias. Study estimates were pooled using a fixed effects sample-weighted meta-analysis approach to calculate an overall estimate and 95% confidence interval (CI). Results on PCI-related toxicity, QoL, and OS were only reported descriptively.

**Results:**

Seven RCTs were included in the meta-analysis. In total, 1,462 patients were analyzed, including 717 patients who received PCI and 745 patients who did not. The risk of developing BM was significantly decreased through PCI (13% reduction, RR 0.33; 95% CI 0.22–0.45). PCI-related toxicity and QoL data were limited. Acute toxicity mostly included fatigue, skin-related toxicity, and nausea or vomiting. Late toxicities such as headache, dyspnea, lethargy, and low grade cognitive impairments were also reported in some of the included RCTs. Results on OS were inconclusive.

**Conclusion:**

The risk of developing BM was reduced in patients who received PCI compared to patients who did not. To implement PCI as the standard treatment for patients with NSCLC, the impact of PCI-related toxicity on QoL should be further investigated, as well as long-term OS. A future individual patient data meta-analysis could produce definitive answers to this clinical question.

## Introduction

Non-small cell lung cancer (NSCLC) is the most important cause of death due to cancer worldwide, and accounts for about 85% of all lung cancers. At present, more than 50% of all patients are diagnosed with adenocarcinoma, less than 10% are diagnosed with large cell cancer and the rest with squamous cell carcinoma. One-third of NSCLC present with locally advanced (stage III) disease, 20% with stage I–II, and the rest have metastases (stage IV) at diagnosis (1).

Non-small cell lung cancer patients frequently develop brain metastases (BM), even though the initial staging with brain CT or MRI was negative. The more advanced the disease stage is, the more frequent BM occur. They are also more frequent in adenocarcinoma than in squamous cell cancer (1). Stage III patients have a BM incidence of approximately 30% (2). With longer overall survival (OS) and better imaging techniques, this percentage might increase. For example, in drive-mutated patients (e.g., EGFR and ALK) with a survival beyond 5 years, this percentage increases to more than 50% (3). BM can lead to neurocognitive disorders, loss of quality of life (QoL), and they are the most important factors influencing patients’ OS (2). Although a radical local treatment of BM may be possible with radiosurgery or resection, the prognosis often remains poor. In order to improve QoL as well as OS, there is an unmet need to prevent the occurrence of BM (4).

Prophylactic cranial irradiation (PCI) was shown to significantly improve OS (5.4% improvement of 3-year OS) in localized small cell lung cancer with complete remission or stable disease after multimodality treatment, as a result of decreasing BM incidence by about 50% (5). Also in patients with NSCLC, several randomized controlled trials (RCTs) studied the value of PCI in the prevention of BM (6–14). However, PCI might deteriorate QoL as a result of neurocognitive decline associated with cranial irradiation. Recently, a randomized phase III trial conducted by the NVALT/DLCRG (14) showed that PCI reduced the incidence of symptomatic BM [7.0% in PCI vs 27.2% in no PCI, hazard ratio 0.25; 95% confidence interval (95% CI) 0.11–0.58]. Therefore, it is time to update the previously published literature and revisit the role of PCI in the prevention of BM in NSCLC patients. Here, we report on the results of a meta-analysis assessing the impact of PCI on the reduction of BM in primary stage I–III NSCLC patients, with PCI-related toxicity, QoL, and OS as secondary endpoints.

## Methods

### Data Collection

Data from published RCTs comparing PCI with non-PCI were sought using electronic database searching between 1980 and December 1, 2017 (PubMed), hand searching (reference checking of individual studies and review articles), and by contacting experts in the field. The following keywords were used as search terms: “Carcinoma, Non-Small-Cell Lung,” “NSCLC,” “Cranial Irradiation,” “Cranial Neoplasms/radiotherapy,” “Brain Metastasis,” “Overall Survival,” and “RCT.” Details of the search strategy and corresponding flow chart can be found in Appendix S1 in Supplementary Material.

### Selection Criteria

Trials were included if they considered a randomized comparison of PCI and non-PCI, enrolled NSCLC patients, excluded patients with recurrent or metastatic disease, and reported results on BM occurrence.

### Quality Assessment

Two investigators (Willem J. A. Witlox and Bram L. T. Ramaekers) independently assessed each RCT for methodological quality using the Cochrane collaboration’s tool for the assessment of risk of bias (15). This tool consists of seven items, including random sequence generation, allocation concealment, blinding of participants and personnel, blinding of outcome assessment, incomplete outcome data, selective reporting, and other sources of bias. Each item was scored “low risk,” “unclear risk,” or “high risk” of bias (Appendix S2 in Supplementary Material).

### Statistical Analysis

Data of the primary endpoint (BM occurrence) was analyzed using Stata/SE 14.2 (16). Relative risk (RR) and accompanying 95% CI of the individual studies were calculated based on the number of events and group totals. Subsequently, the estimates were pooled using a fixed effects sample-weighted meta-analysis approach to calculate an overall estimate and 95% CI. Heterogeneity of the studies was tested using chi-square- and *I*^2^-tests (15). Publication bias and small study effects were assessed by visual inspection of funnel plots and performing Egger’s test, respectively (17, 18). If Egger’s test is significant, a sensitivity analysis will be performed excluding small studies (weight < 10%).

Results on PCI-related toxicity, QoL, and OS will only be reported descriptively.

## Results

### Literature Search and Quality Assessment of Publications

**Figure 1:**
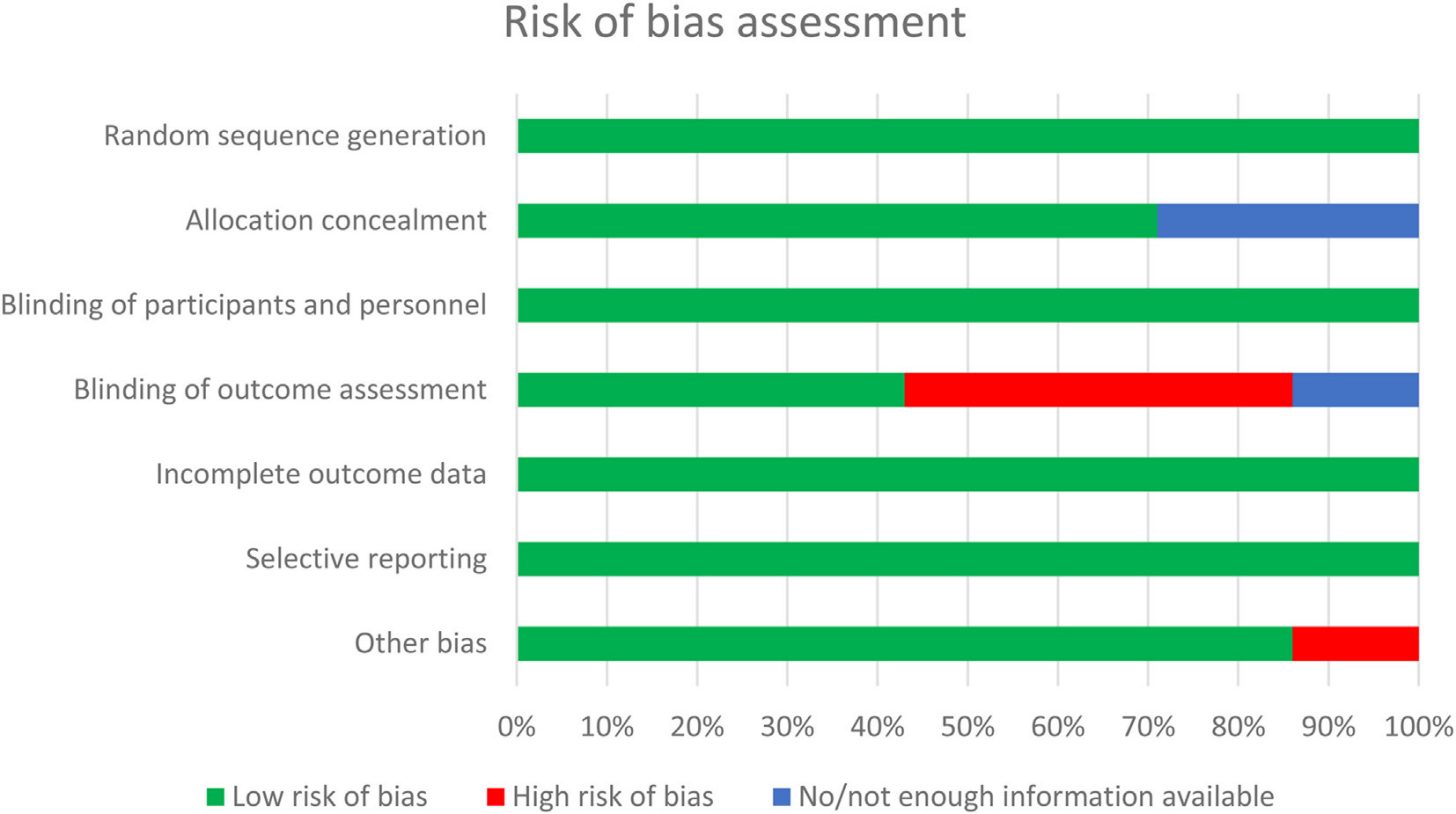
Methodological quality assessment of the included randomized controlled trials evaluating the effect of prophylactic cranial irradiation on brain metastases in non-small cell lung cancer.

The electronic literature search yielded 360 unique publications. Another two publications were identified through hand searching and contacting experts. After screening of titles and/or abstracts, 354 trials were excluded. One RCT (12) was excluded after reading the full text, because local treatment was different between both arms. Methodological quality of the remaining seven RCTs was checked and most of the items were at “low risk” of bias (Figure 1). Three studies (6–8) did only perform a brain scan when indicated by a change in neurological status of the patients, without adequately defining how neurological status was assessed. Therefore, the reviewers judged these studies to be at high risk of introducing bias in assessing the outcome. The reviewers suspected possible selection bias in the study of Cox et al. (6), because randomized patients were excluded from evaluation, which is not in accordance with the intention to treat principle. Although blinding of participants and personnel was not performed in any of the included studies, based on the nature of the intervention, this item was judged by the reviewers as low risk of introducing bias (Appendix S2 in Supplementary Material).

### Characteristics of the Included Trials

Characteristics of the seven included trials are listed in Table 1 below. There were little differences between the selected patient groups of the trials. Four studies (9, 10, 13, 14) included stage III NSCLC patients only, two studies (7, 8) included stage I, II, and III patients, and in one study (6) staging was unclear. For treatment of the primary tumor, two trials (6, 14) treated their patients with radiotherapy alone, one trial (11) used chemo-radiotherapy, and the four remaining trials used either combinations of chemo-radiotherapy and radiotherapy alone (7, 9, 10), chemotherapy and surgery (13), or radiotherapy and surgery (8). Brain imaging was mainly done by a radionuclide scan in two studies (6, 7). One study (8) used CT scans, three more recent studies (11, 13, 14) used MRI and in one study (9, 10) the technology of brain imaging was unclear. Dosing of cranial irradiation ranged from 20 to 37.5 Gy (10 fractions of 2 Gy to 15 fractions of 2.5 Gy).

**Table 1:** Study characteristics of the included RCTs evaluating PCI.

Study	Year of publication	Local treatment	Stage	Brain imaging	PCI dose	Primary endpoint	*N*^a^	Systematic Follow-up for BM
VALG	1981	RT alone	All^b^	Radionuclide scan	10 Gy × 2 Gy	BM rate	281	No

MDACC	1984	Chemo-RT	I–III	Radionuclide scan/CT scan	10 Gy × 3 Gy	CNS metastases rate	97	No
		RT alone						

RTOG 8403	1991	RT alone	I–III	CT scan	10 Gy × 3 Gy	Time to BM	187	No
		Surgery and RT						

SWOG	1998	Chemo-RT	III	Unclear	15 Gy × 2–2.5 Gy	OS rate	226	Not reported
		RT alone						

RTOG 0214	2012	Chemo-RT	III	MRI scan	15 Gy × 2 Gy	OS rate	340	Yes

Li	2015	Surgery-chemo	IIIA	MRI scan	10 Gy × 3 Gy	DFS	156	Yes

NVALT-11	2018	RT alone	III	CT scan/MRI scan	18 Gy × 2 Gy/12 Gy × 2.5 Gy/10 Gy × 3 Gy	Symptomatic BM rate	175	Yes

*^a^Number of eligible patients*.

*^b^All inoperable patients; stage not clear*.

*PCI, prophylactic cranial irradiation; RT, radiotherapy; Chemo; chemotherapy; BM, brain metastases; CNS, central nervous system; OS, overall survival; DFS, disease-free survival; RCTs, randomized controlled trials*.

### Incidence of BM After PCI

**Figure 2:**
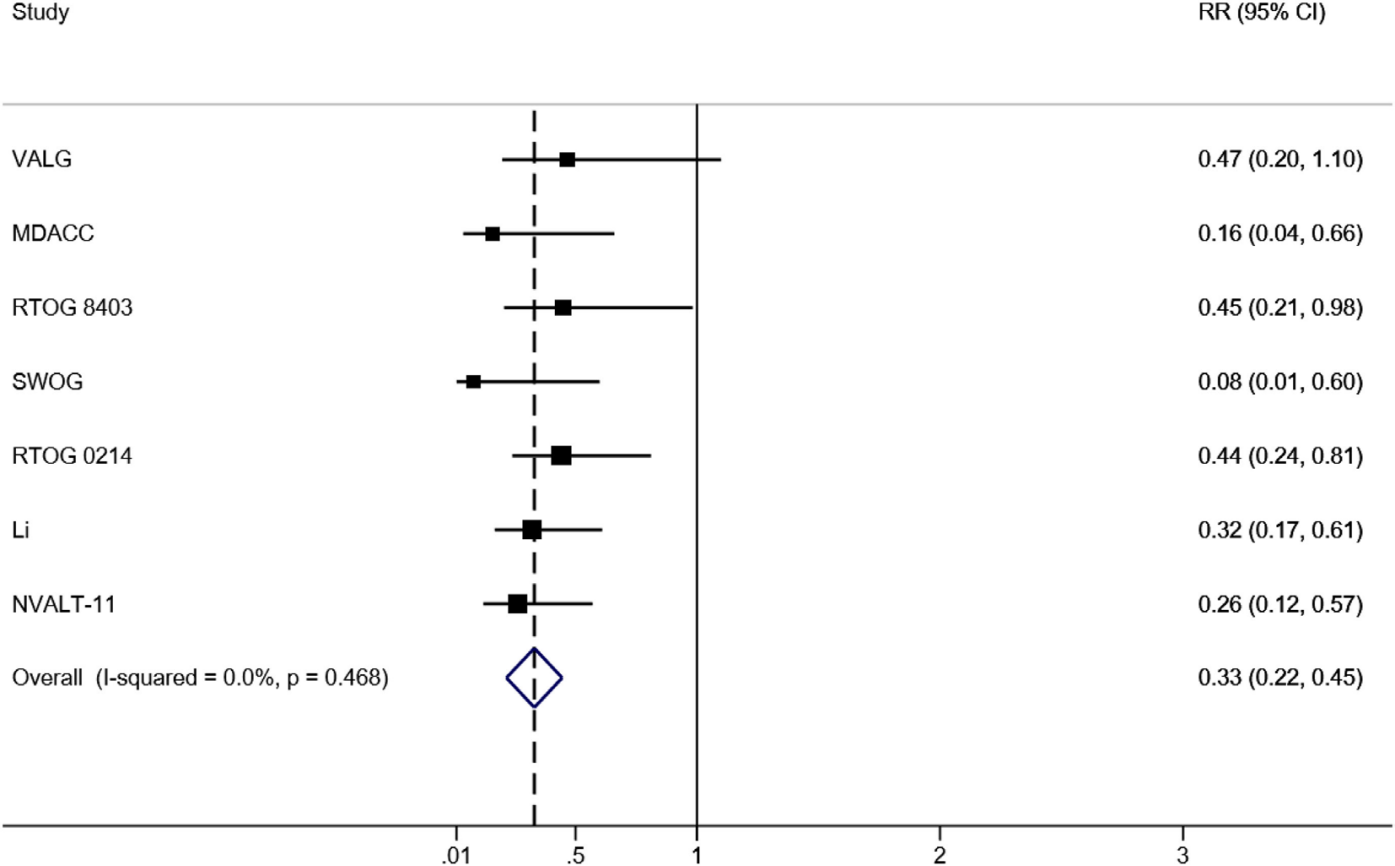
Forrest plot of the meta-analysis of RCTs evaluating the effect of PCI on BM in NSCLC. Abbreviations: RR, relative risk; 95% CI, 95% confidence interval; RCTs, randomized controlled trials; PCI, prophylactic cranial irradiation; BM, brain metastases; NSCLC, non-small cell lung cancer.

**Figure 3:**
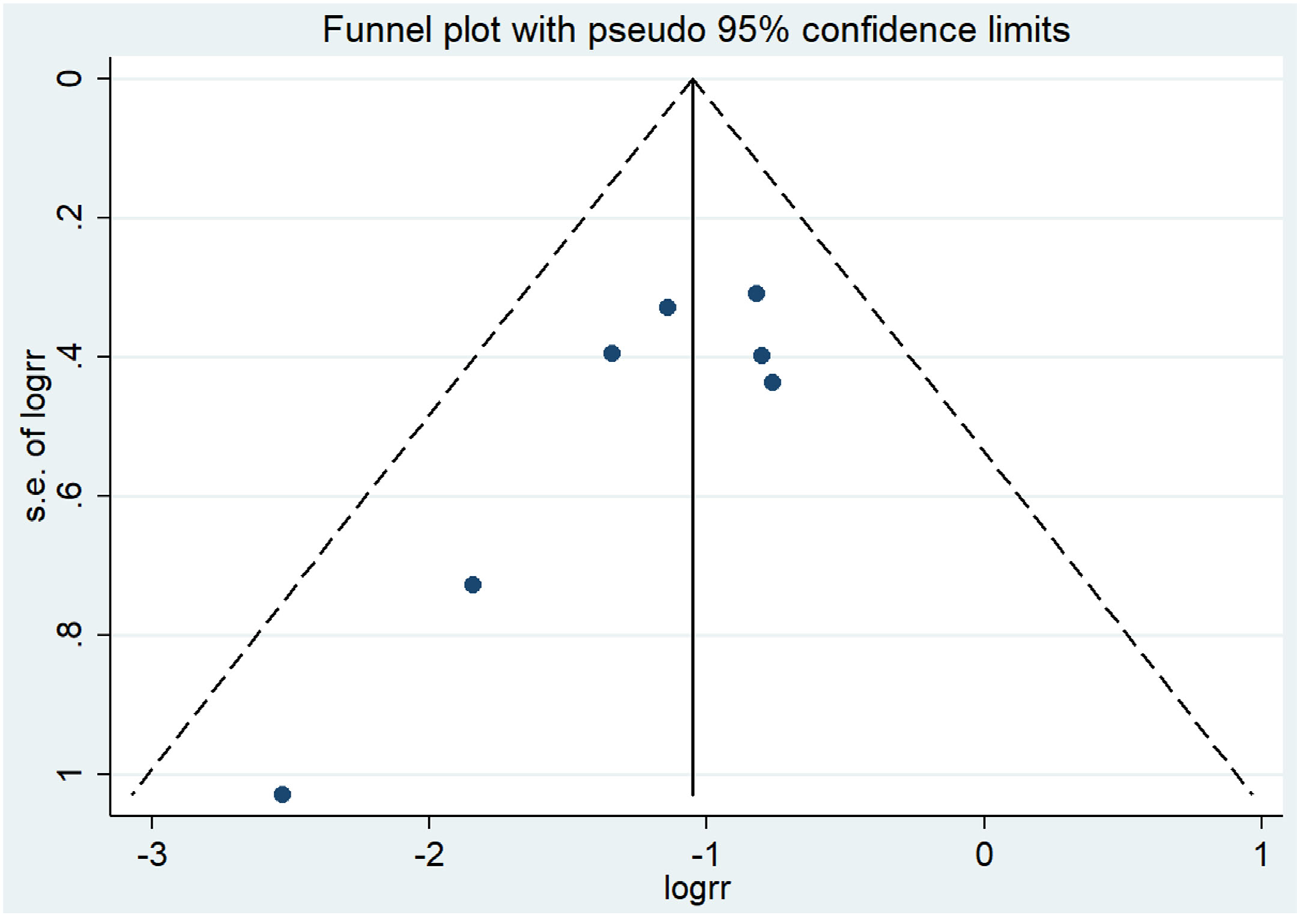
Results of the funnel plot of RCTs evaluating the effect of PCI on BM in NSCLC. Abbreviations: logrr: logarithm of the relative risk; RCTs, randomized controlled trials; PCI, prophylactic cranial irradiation; BM, brain metastases; NSCLC, non-small cell lung cancer.

Taken all RCTs together, in total, 1,462 patients were analyzed, including 717 patients who received PCI and 745 patients who did not. The BM incidence in the PCI arm ranged from 0.9 to 12.3%, and from 11.0 to 30.7% in the non-PCI arm (Table 2). The overall effect estimate of the impact of PCI on the occurrence of BM is presented in Figure 2. The risk of developing BM was significantly decreased in the PCI arm compared to no PCI (13% reduction, RR 0.33; 95% CI 0.22–0.45). Heterogeneity across the studies was low (*I*^2^ = 0%; *p* = 0.468). Furthermore, Egger’s test indicated that smaller studies showed larger effect sizes (*p* = 0.048), which is also reflected in the asymmetric funnel plot (Figure 3). Nevertheless, results of the sensitivity analysis excluding small studies (7, 9, 10) (weight < 10%) were similar (RR 0.38; 95% CI 0.24–0.51).

**Table 2:** Data on BM events [(a)symptomatic BM occurrence] and incidence of the included RCTs evaluating PCI.

	PCI			No PCI			
Study	Events	Total	Incidence (%)	Events	Total	Incidence (%)	Weight (%)
VALG	7	136	5.1	16	145	11.0	10.5
MDACC	2	46	4.3	14	51	27.5	9.0
RTOG 8403	8	93	8.6	18	94	19.1	12.2
SWOG	1	111	0.9	13	115	11.3	8.7
RTOG 0214	13	163	8.0	32	177	18.1	20.9
Li	10	81	12.3	29	75	38.7	20.5
NVALT-11	7	87	8.0	27	88	30.7	18.3
Total	48	717		149	745		

*PCI, prophylactic cranial irradiation; BM, brain metastasis; RCTs, randomized controlled trials*.

### PCI-Related Toxicity, QoL, and OS

Few trials reported on PCI-related toxicity and QoL with most details in the study of Gore et al. (11) (Table 3). Acute toxicity mostly included fatigue, skin-related toxicity, and nausea or vomiting. Late toxicities such as headache, dyspnea, and lethargy were also reported in some of the included RCTs. Low grade (1 and 2) memory impairments and cognitive disturbances were only reported in the study of De Ruysscher et al. (14). Results reporting on QoL were limited, and no significant differences were observed between both arms.

**Table 3:** RCTs evaluating PCI-related toxicity and QoL in NSCLC.

Study	PCI dose (Gy/fraction)	Neuropsychological test	Evaluation after PCI	Impairment after PCI	QoL instruments and results
MDACC	30/10	Not reported	Not reported	Acute toxicity: one patient developed transient memory loss for 2.5 weeks	Not reported
				Late toxicity: none	

RTOG 8403	30/10	Neurological physical examinations	Intervals of 3 months	Acute toxicity: epilation and skin reactions	Not reported
				Late toxicity: none	

RTOG 0214	30/15	MMSE ADLS HVLT	At 3, 6, 12, 18, 24, 30, 36, and 48 months, and then yearly	Acute toxicity: constitutional (grade 1 and 2), gastrointestinal (grade 1), dermatologic (grade 2), hematologic (grade 3), fatigue (grade 3), dyspnea (grade 3), ataxia (grade 3), depression (grade 3 and 4)	EORTC QLQ-C30 and EORTC QLQ-BN20
				Late toxicity: dyspnea, syncope, weakness, fatigue (all grade 3)	Global health status/QoL was similar between both arms

Li	30/10	CTC-AE RTOG/EORTC-LRMSS	First 2 years every 3 months, every 6 months thereafter	Acute toxicity: headache (grade 1, 2, and 3), nausea or vomiting (grade 1 and 2), fatigue (grade 1, 2, and 3), skin toxicity (grade 1 and 2), insomnia (grade 2)	FACT-L questionnaire
				Late toxicity: mild, moderate, and severe headache, slight or great lethargy, skin atrophy, fatigue	No significant differences were noted in deterioration rate for QoL and symptoms between the two groups

NVALT-11	36/18 30/12 30/10	CTC-AE	4 weeks, 3, 6, 12, 24, and 36 months	Memory impairment (grade 1 and 2), cognitive disturbance (grade 1 and 2), alopecia, fatigue, headache	EORTC QLQ-C30 EORTC QLQ-BN20 EuroQol 5D Results not reported

*PCI, prophylactic cranial irradiation; QoL, quality of life; MMSE, Mini Mental Status Exam; ADLS, Activities of Daily Living Scale; HVLT, Hopkins Verbal Learning Test; CTC-AE, Common Terminology Criteria Adverse Events; RTOG/EORTC-LRMSS, RTOG/ERTOC late radiation morbidity scoring schema; EORTC QLQ-C30, EORTC quality of life questionnaire-C30; EORTC QLQ-BN20, EORTC quality of life questionnaire-BN20; FACT-L questionnaire, Functional Assessment of Cancer Therapy-Lung questionnaire; NSCLC, non-small cell lung cancer; RCTs, randomized controlled trials*.

In addition to BM occurrence, all included trials, except for the trial of Cox et al. (6), also reported on PCI-related OS in NSCLC (Table 4). Nearly all studies only report on short-term survival, and most trials did not use contemporary staging and systemic therapy. Taking these shortcomings in mind, no statistically significant OS difference was found between the PCI arm and no PCI arm, except for the study of Miller et al. (9, 10), which showed a significant OS benefit in favor of the no PCI arm (*p* = 0.004).

**Table 4:** RCTs evaluating PCI-related OS in NSCLC.

Study	Median follow-up in months	Median OS in months (PCI vs no PCI)	*p*-Value
VALG	Not reported	Not reported	Not reported
MDACC	13.6 (PCI), 12.7 (no PCI)	60.3 vs 56.3	Not significant
RTOG 8403	Not reported	8.4 vs 8.3	0.36
SWOG	Not reported	8.0 vs 11.0	Significant
RTOG 0214	23.8	25.8 vs 24.8	0.86
Li	68.1 (PCI), 65.2 (no PCI)	31.2 vs 27.4	0.31
NVALT-11	53.3	24.2 vs 21.9	0.56

*OS, overall survival; PCI, prophylactic cranial irradiation; NSCLC, non-small cell lung cancer; RCTs, randomized controlled trials*.

## Discussion

This study aimed to review published literature and revisit the role of PCI in the prevention of BM in NSCLC. The analysis included seven RCTs, involving 1,462 NSCLC patients in total. The current meta-analysis shows that the risk of developing BM was reduced in patients who received PCI compared to patients who did not (RR 0.33; 95% CI 0.22–0.45).

Previously published results from reviews that investigated the role of PCI in the prevention of BM in NSCLC are in line with our findings. The most recently published meta-analyses of Sun et al. (4) and Xie et al. (19), evaluating the impact of PCI on BM occurrence in NSCLC, showed highly significant results in favor of PCI. However, these reviews could not evaluate the recent RCTs of Li et al. (13) and De Ruysscher et al. (14). Including the two most recent RCTs does not only add to the sample size of our meta-analysis, but the proper methodological quality and the use of more advanced brain imaging methods also add value to the conclusiveness of our results. Furthermore, unlike our study, the RCT of Pottgen et al. (12) was included in these meta-analyses. Local treatment was different between the two arms in this study (primary curative resection followed by postoperative thoracic radiation therapy vs chemotherapy and concurrent chemo-radiotherapy followed by thoracic surgery). Therefore, we judged that it was not possible to assess the impact on prevention of BM attributable to PCI.

Although this is the largest, most recent review incorporating all available evidence from RCTs on the impact of PCI on the prevention of BM in NSCLC, there are also limitations. Egger’s test and visual inspection of the funnel plot indicated the presence of publication bias. However, sensitivity analysis showed that excluding smaller studies from the meta-analysis did not much alter the results. Furthermore, assessment of the methodological quality of the included studies showed that for some items risk of bias was high. Nevertheless, most of these high bias risk items could be found in the studies (7, 9, 10) that were excluded in the sensitivity analysis, and results remained largely similar to the original results.

The meta-analysis showed that the BM incidence was lower in patients who received PCI, but few trials also reported that PCI could cause toxicity resulting in a decline in QoL. Most occurring acute toxicities were fatigue, skin-related toxicity, and nausea or vomiting. Toxicities occurring on longer term were headache, dyspnea, and lethargy. In addition, the study of De Ruysscher et al. (14) also reported low-grade memory and cognitive functioning impairments. Therefore, the indications of PCI should be considered in the light of its potential (neuro)toxicity. QoL data were limited and not significantly different between the groups, no short-term OS benefit was shown, and the influence of PCI on long-term OS should be further investigated. It is necessary to further study the role of PCI in relation to neurocognitive decline and thus deterioration of QoL, and whether PCI could improve patients’ long-term OS. In the era of more effective targeted therapy and immunotherapy, when extracranial disease is better controlled and patients are living longer, there may be increased importance of PCI. Moreover, hippocampal sparing techniques and medications such as memantine could be interesting future areas of research as alternatives to reduce toxicity and thus loss of QoL. Other areas of future research might include the role of MRI surveillance in combination with radical local treatment such as stereotactic radiosurgery or whole-brain radiotherapy. However, studies of the EORTC and RTOG showed that cure remains elusive in the overwhelming majority of these patients (20, 21).

## Conclusion

The risk of developing BM was reduced in patients who received PCI compared to observation. To implement PCI as the standard treatment for patients with NSCLC, the impact of PCI on toxicity and QoL should be further investigated, as well as the impact on long-term OS. A future individual patient data meta-analysis with updated long-term OS could potentially produce definitive answers to these clinical questions.

## Author Contributions

WW wrote the initial draft of this review, with edits and revisions from all other authors, and especially BR and DR.

## Conflict of Interest Statement

LH: Consulting or Advisory Role: Boehringer Ingelheim and BMS. Fees for lectures: MSD, Astra Zeneca, Roche. Research grant: Roche. A-MD: Consulting or Advisory Role: Genentech (Inst), MSD Oncology (Inst), AstraZeneca (Inst), Pfizer, Eli Lilly (Inst), Boehringer Ingelheim (Inst), Bristol-Myers Squibb, Clovis Oncology (Inst). DR: Consulting or Advisory Role: Bristol-Myers Squibb (Inst), Genentech (Inst), Merck Serono (Inst), AstraZeneca (Inst), Celgene (Inst). Research Funding: Bristol-Myers Squibb (Inst). The remaining authors declare that the research was conducted in the absence of any commercial or financial relationships that could be construed as a potential conflict of interest.
