# Supplementary material for: The Prevention of Brain Metastases in Non-Small Cell Lung Cancer by Prophylactic Cranial Irradiation
Source: Front Oncol. 2018 Jul 26;8:241. doi: 10.3389/fonc.2018.00241 (PMC6070634; doi:10.3389/fonc.2018.00241)
Supplement: Supplementary file 1 [file data_sheet_1.docx]

**Appendix 1**

**MEDLINE electronic search strategy**

| **Search** | **Query** | **Items found** |
| --- | --- | --- |
| **Patient** | | |
| #1 | Carcinoma, Non-Small-Cell Lung[MeSH Terms] | 42,471 |
| #2 | NSCLC*[Title/Abstract] | 32,064 |
| **#**3 | (lung*[Title/Abstract]) OR (bronch*[Title/Abstract]) | 692,736 |
| #4 | (carcino*[Title/Abstract]) OR (cancer*[Title/Abstract]) OR (tumor*[Title/Abstract]) OR (tumour*[Title/Abstract]) OR (malignan*[Title/Abstract]) OR (oncol*[Title/Abstract]) | 2,837,970 |
| #5 | #1 OR #2 OR (#3 AND #4) | 254,217 |
| **Intervention** | | |
| #6 | Cranial Irradiation[MeSH Terms] | 4,854 |
| #7 | Brain Neoplasms/radiotherapy[MeSH Terms] | 12,181 |
| #8 | (brain*[Title/Abstract]) OR (cran*[Title/Abstract]) | 1,007,160 |
| #9 | (carcino*[Title/Abstract]) OR (cancer*[Title/Abstract]) OR (tumor*[Title/Abstract]) OR (tumour*[Title/Abstract]) OR (malignan*[Title/Abstract]) OR (oncol*[Title/Abstract]) OR (metasta*[[Title/Abstract]) | 2,837,971 |
| #10 | (Radiotherapy[MeSH Terms]) OR (Radioth*[Title/Abstract]) OR (Radiat*[Title/Abstract]) | 518,712 |
| #11 | #6 OR #7 OR (#8 AND #9 AND #10) | 31,341 |
| **Outcome** | | |
| #12 | Survival Analysis[MeSH Terms] | 243,741 |
| #13 | Radiation Effects[MeSH Terms] | 71,206 |
| #14 | Brain/Radiation Effects[MeSH Terms] | 8,646 |
| #15 | Survival[MeSH Terms] | 4,433 |
| #16 | Mortality[MeSH Terms] | 334,306 |
| #17 | Survival Rate[MeSH Terms] | 150,629 |
| #18 | Survival*[Title/Abstract] | 782,165 |
| #19 | #12 OR #13 OR #14 OR #15 OR #16 OR #17 OR #18 | 1,162,700 |
| **Design** | | |
| #20 | RCT*[Title/Abstract] | 34,109 |
| #21 | random*[Title/Abstract] | 944,737 |
| #22 | #20 OR #21 | 949,604 |
| #23 | #5 AND #11 AND #19 AND #22 | **360** |


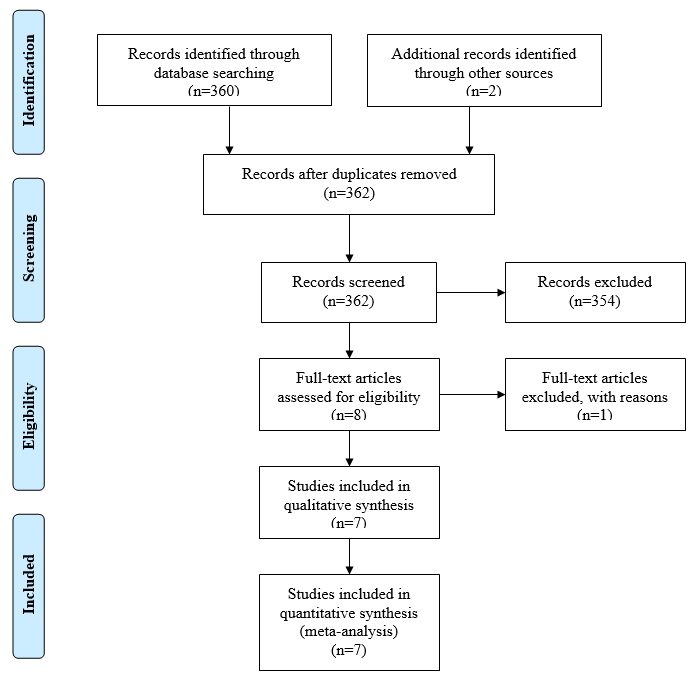


**Flowchart of the trial selection**

**Appendix 2**

**Methodological quality of the included RCTs evaluating the effect of PCI on BM in NSCLC**

| **Study** | **Random sequence generation** | **Allocation concealment** | **Blinding of participants and personnel** | **Blinding of outcome assessment** | **Free of Incomplete outcome data** | **Free of Selective reporting** | **Free of other bias** |
| --- | --- | --- | --- | --- | --- | --- | --- |
| **VALG** | V | V | V | X | V | V | X |
| **MDACC** | V | ? | V | X | V | V | V |
| **RTOG8403** | V | V | V | X | V | V | V |
| **SWOG** | V | ? | V | ? | V | V | V |
| **RTOG0214** | V | V | V | V | V | V | V |
| **Li** | V | V | V | V | V | V | V |
| **NVALT-11** | V | V | V | V | V | V | V |

V = low risk of bias

X = high risk of bias

? = no/not enough information available
